# CARDIOVASCULAR RNA MARKERS AND ARTIFICIAL INTELLIGENCE MAY IMPROVE COVID-19 OUTCOME: POSITION PAPER FROM THE EU-CardioRNA COST Action CA17129

**DOI:** 10.1093/cvr/cvab094

**Published:** 2021-04-11

**Authors:** Lina Badimon, Emma L Robinson, Amela Jusic, Irina Carpusca, Leon J de Windt, Costanza Emanueli, Péter Ferdinandy, Wei Gu, Mariann Gyöngyösi, Matthias Hackl, Kanita Karaduzovic-Hadziabdic, Mitja Lustrek, Fabio Martelli, Eric Nham, Ines Potočnjak, Venkata Satagopam, Reinhard Schneider, Thomas Thum, Yvan Devaux, EU-CardioRNA COST Action CA17129

**Affiliations:** 1 Cardiovascular Science Program-ICCC, IR-Hospital de la Santa Creu i Santa Pau, Ciber CV, Autonomous University of Barcelona, Barcelona; 2 Department of Cardiology, School for Cardiovascular Diseases, Faculty of Health, Medicine and Life Sciences, Maastricht University, Maastricht, The Netherlands; Division of Cardiology, Department of Medicine, University of Colorado Anschutz Medical Campus, Aurora, CO, USA; 3 Cardiovascular Research Unit, Luxembourg Institute of Health, Strassen, Luxembourg; 4 Department of Molecular Genetics, Faculty of Science and Engineering; Faculty of Health, Medicine and Life Sciences, Maastricht University, Maastricht, The Netherlands; 5 National Heart & Lung Institute, Faculty of Medicine, Imperial College London, London, UK; 6 Cardiometabolic Research Group and MTA-SE System Pharmacology Research Group, Department of Pharmacology and Pharmacotherapy, Semmelweis University, Budapest, Hungary; Pharmahungary Group, Szeged, Hungary; 7 Luxembourg Center for Systems Biomedicine, University of Luxembourg, Esch sur Alzette, Luxembourg; 8 Department of Cardiology, Medical University of Vienna, Vienna, Austria; 9 TAmiRNA GmbH, Vienna, Austria; 10 Faculty of Engineering and Natural Sciences, International University of Sarajevo, Bosnia and Herzegovina; 11 Department of Intelligent Systems, Jozef Stefan Institute, 1000e Ljubljana, Slovenia; 12 Molecular Cardiology Laboratory, IRCCS Policlinico San Donato, San Donato Milanese, 20097 Milan, Italy; 13 University of Zagreb School of Medicine, Zagreb, Croatia; 14 Institute for Clinical Medical Research and Education, University Hospital Centre Sisters of Charity, Zagreb, Croatia; 15 Institute of Molecular and Translational Therapeutic Strategies (IMTTS), Fraunhofer Institute for Toxicology and Experimental Medicine, Hannover, Germany, and REBIRTH Center for Translational Regenerative Medicine, Hannover Medical School, Hannover, Germany

**Keywords:** biomarkers, artificial intelligence, RNAs, genomics

## Abstract

The coronavirus disease 2019 (COVID-19) pandemic has been as unprecedented as unexpected, affecting more than 105 million people worldwide as of February 8th, 2020 and causing more than 2.3 million deaths according the World Health Organization. Not only affecting the lungs and provoking acute respiratory distress, severe acute respiratory syndrome coronavirus 2 (SARS-CoV-2) is able to infect multiple cell types including cardiac and vascular cells. Hence a significant proportion of infected patients develop cardiac events such as arrhythmias and heart failure. Patients with cardiovascular comorbidities are at highest risk of cardiac death. To face the pandemic and limit its burden, health authorities have launched several fast track calls for research projects aiming to develop rapid strategies to combat the disease, as well as longer-term projects to prepare for the future. Biomarkers have the possibility to aid in clinical decision making and tailoring healthcare in order to improve patient quality of life. The biomarker potential of circulating RNAs has been recognized in several disease conditions, including cardiovascular disease. RNA biomarkers may be useful in the current COVID-19 situation. The discovery, validation and marketing of novel biomarkers, including RNA biomarkers, require multi-centre studies by large and interdisciplinary collaborative networks, involving both the academia and the industry. Here, members of the EU-CardioRNA COST Action CA17129 summarize the current knowledge about the strain that COVID-19 places on the cardiovascular system and discuss how RNA biomarkers can aid to limit this burden. They present the benefits and challenges of the discovery of novel RNA biomarkers, the need for networking efforts and the added value of artificial intelligence to achieve reliable advances.

## Introduction: SARS-CoV-2 in 2020

The effect of the coronavirus disease 2019 (COVID-19) pandemic on the cardiovascular system is alarming. More research focusing on the collateral damage associated with COVID-19 infection is needed. COVID-19 causes pneumonia with multi-organ disease. Infection can be asymptomatic or may cause a wide spectrum of symptoms, from mild upper respiratory tract infection to life-threatening sepsis with generalized endothelial damage, inflammation and thrombosis. COVID-19 first emerged in December 2019 in Wuhan, China, and as of February 8th 2020 has affected people in more than 200 countries, with more than 105 million identified cases and with over 2.3 million confirmed deaths (WHO Coronavirus Disease Dashboard). It is clear that one of the causes for the significant differences in the severity of symptoms and mortality may derive from patient susceptibility to infection. Moreover, a significant proportion of COVID-19 survivors suffer cardiovascular damage. As such, there is a clinical need for novel biomarkers which would aid in the identification of patients at risk of suffering a severe form of the disease or that may identify those patients prone to develop collateral damage in the vascular, cardiac and cerebrovascular systems that may jeopardize their future wellbeing. We need to investigate and innovate to detain the next pandemic wave of COVID-related cardiovascular disease.

To face the pandemic and limit its medical, -social and economic burden, health authorities have launched several Fast Track calls for research projects aiming to develop rapid strategies to combat the disease, as well as longer-term projects to learn and draw lessons from the current pandemic and prepare for the future [1].

A myriad of potential biomarkers of COVID-19, for both diagnostic and prognostic purposes, have been highlighted in an extremely high number of published articles within the few months following the beginning of the pandemic. Although it is difficult to identify from all these reports the most relevant biomarkers with serious translational potential, artificial intelligence approaches could constitute a key component of such endeavours. Cardiovascular and blood RNA markers, coupled with artificial intelligence methods, represent a still poorly explored yet rich reservoir of novel biomarkers with some potential to aid in personalizing healthcare of COVID-19 patients. Recent single-cell RNA sequencing experiments support this assumption [2].

## Epidemiology of SARS-CoV-2 and cardiovascular disease

SARS-CoV-2 infection affects mostly the ageing population with pre-existing cardiovascular diseases, such as coronary artery diseases, heart failure or respiratory failure of any origin. Moreover, individuals with pre-existing risk factors for cardiovascular disease or with co-morbidities affecting the cardiovascular system, are at high-risk for worse clinical outcome during the infection [3]. Frequent involvement of cardiovascular comorbidities is detected in patients with SARS-CoV-2 infection and up to 33% of hospitalized patients with a COVID-19 positive test have cardiac injury [4] evidenced by elevated cardiac troponin I and troponin T levels. These patients are prone to develop acute heart failure and have a high (up to 44.4% reported) burden of arrhythmias [5-8]. Patients with SARS-CoV-2 infection and acute cardiac injury have a substantially higher rate of in-hospital mortality (up to 71.2%), as compared with the mortality of patients with SARS-CoV-2 infection and no evidence of cardiac injury [3, 7]. Patients with pre-existing heart failure and SARS-CoV-2 infection have a two-fold higher risk of 30-day mortality as compared to patients without pre-exiting heart failure and SARS-CoV-2 infection, independently of the category of heart failure (reduced, mid-range, or preserved ejection fraction) [9]. Multi-organ failure due to hypoxia caused by respiratory failure, acute kidney injury, electrolyte disturbances, systemic inflammation and cytokine storm contribute to the cardiac injury in patients with SARS-CoV-2. The cytokine storm seems to contribute to a large extent to cardiac and vascular events. However, there are reports asking for a more concise definition of the cytokine storm and its real impact in the pathogenesis of the infection [10-12]. Altered coagulation may lead to thrombotic complications including microthrombosis, microvascular damage, and generalized thromboembolic disorder. Recent empirical drugs against OVID-19 such as chloroquine, antiviral or anti-rheumatic drugs, monoclonal antibodies or antibiotics may also aggravate cardiovascular symptoms by prolonging QT interval leading to arrhythmias, or resulting in drug-induced cardiomyopathies or cardiotoxicity [4]. Since SARS-CoV-2 has a strong affinity for the angiotensin-converting enzyme 2 (ACE2) cell receptor, it was plausible to assume that antihypertensive treatment with ACE inhibitors or angiotensin receptor blockers (ARBs) might aggravate the disease. To date, however, no clinical evidence can confirm this assumption, thus ACE inhibitor and ARB treatments continue to be administered to SARS-CoV-2 positive patients [13, 14]. The lockdown regulations and subsequent closure of out-patient clinics has led to major reorganizational efforts of the management of patients with cardiovascular disease. A paradoxical decrease of documented acute myocardial infarction has also been observed, which could be attributed to the lack of preventive control of patients with chest pain, and the self-quarantining of the patients fearing from the risk of nosocomial infection [15].

## Pathophysiology of SARS-CoV-2 infection phases; effects on the heart

SARS-CoV-2, a member of the family of coronaviruses, is an enveloped, positive-sense, single-stranded RNA virus that is able to infect various host species [16]. Among the viral–encoded proteins, the SARS-CoV-2 spike (S) transmembrane glycoprotein protrudes from the viral surface and is essential for target cell binding and infection. ACE2 has been identified as the SARS-CoV-2 receptor [17-20] and ACE2 is highly expressed in the lung, heart, ileum, kidney and bladder [21]. The majority of adaptive immune cells that invade the infected lung tissue consist of T cells, since a proportional decrease in circulating T cells has been observed in COVID-19 patients. IL-8 and IL-6, recognized chemo-attractants for T cells and neutrophils, are produced by SARS-CoV-2-compromised lung epithelial cells (Figure 1a) [22]. As neutrophils function in adaptive immunity but can also provoke further damage to the lung, these cells are regarded as double-edged swords in the context of COVID-19 [23]. Circulating monocytes are attracted from the circulation by granulocyte macrophage colony stimulating factor that is produced by local T cells in infected tissue. In addition, elevated CD14+CD16+ inflammatory monocytes producing high levels of IL-6 are found in COVID-19 patients, suggesting that also monocytes actively contribute to the systemic inflammatory response. Finally, thrombosis and pulmonary embolism are commonly observed in severely-ill COVID-19 patients (Figure 1b), likely indicating the presence of significant endothelial injury and microvascular permeability, which may further exacerbate viral invasion.

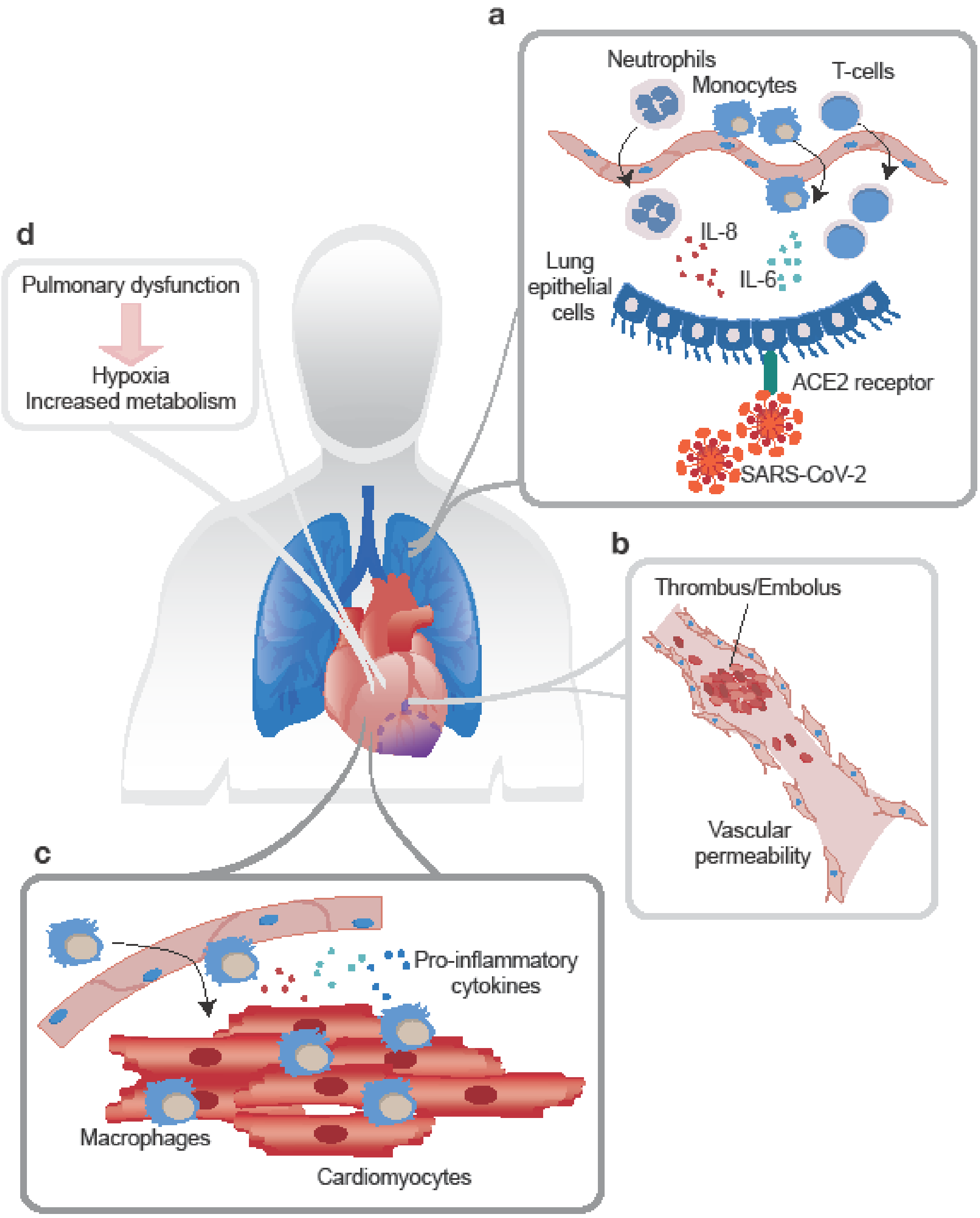

**Figure 1**. Disease mechanisms of SARS-CoV-2 infection.

The symptoms of COVID-19 patients are heterogeneous, ranging from minimal symptoms to significant hypoxia with acute respiratory distress, shock, coagulation dysfunction, and multi-organ involvement, including acute kidney injury, encephalopathy, myocardial injury and heart failure. Indeed, epidemiological, clinical and biological evidence shows a clear cardiac involvement in COVID-19 patients, due to direct myocardial infection and injury and/or to indirect mechanisms, linked to the underlying pathophysiology of the disease [24].

In keeping with a direct effect on heart function of SARS-CoV-2 (Figure 1c), its receptor ACE2 is expressed by cardiomyocytes, fibroblasts, endothelial cells, pericytes, macrophages and the epicardial fat [21]. Moreover, ACE2 levels are increased in failing hearts and its high expression in arterial vascular cells of fibrotic lungs may facilitate the bloodstream spreading of SARS-CoV-2 [25]. Cardiomyocytes derived from human induced pluripotent stem cells can be infected efficiently by SARS-CoV-2 [26, 27]. The SARS-CoV-2 genome has been identified in endomyocardial biopsies of patients with suspected myocarditis [28]. However, while cardiomyocyte damage was present, no viral particles were detected in cardiomyocytes and endothelium, suggesting that the particles were due to infected macrophage migration. Thus, direct myocardial infection may not be the main mechanism of myocardial damage explaining the frequently observed troponin increases. The release of inflammatory cytokines (Figure 1c), a hallmark of severe COVID-19, can also lead to a form of myocarditis resembling Takotsubo syndrome [29]. Moreover, the pro-thrombotic state of COVID-19 patients, associated to D-dimers increase, may lead to microvascular dysfunction, coronary thrombosis or embolism (Figure 1b) [30]. Along with the pro-coagulant profile of patients with COVID-19 [31], other forms of stress may facilitate cardiomyopathy occurrence, such as hypoxemia caused by respiratory dysfunction, endothelial dysfunction leading to small arterial obliteration [28], and the increased metabolic demands (Figure 1d).

## Treatments: what is available, what is needed

### *Remdesivir*

*Remdesivir* is the first medicinal product for human use for the treatment of COVID-19 which was granted a conditional marketing authorization of the European Parliament and of the Council [32]. It is a nucleotide analogue with a broad-spectrum antiviral activity. The European Medicines Agency, specifically the Committee for Medicinal Products for Human Use, has granted a conditional marketing authorization to Veklury (*remdesivir*) for the treatment of COVID-19 in adults and adolescents with pneumonia who require supplemental oxygen ($O_2$) [33]. The recommendation of *remdesivir* is mainly based on the results of the Adaptive COVID-19 Treatment Trial (ACTT)-1 sponsored by the US National Institute of Allergy and Infectious Diseases, and supporting data from other studies on *remdesivir* [33, 34]. According to the ACTT-1 study, patients in the *remdesivir* group had a shorter time to recovery than patients in the placebo group (median 10 vs.15 days) [35]. Kaplan–Meier estimates of mortality at day 29 were 11.4% in the *remdesivir* group and 15.2% in the placebo group (hazard ratio 0.73; 95% CI 0.52 - 1.03) [35]. The Food and Drug Administration (FDA) issued an emergency use authorization [36]. The use o*f remdesivir* has shown shortening of recovery time in severe patients with $O_2$ saturation ≤ 94%, and cases requiring supplemental $O_2$, mechanical ventilation, or extracorporeal membrane oxygenation [37, 38]. It is recommended to start the treatment on day 1 with 200 mg infusion, followed by 100 mg infusion daily for at least 4 days and maximum 9 days [33]. According to the WHO SOLIDARITY trial (results in preprint), death rate ratios for *remdesivir* are RR=0.95 (95% CI 0.81-1.11, p=0.50) [39]. Comparative results from other studies are shown in Table 1. Overall, remdesivir, while improving time to recovery in patients with mild symptoms in ACTT1 trial, fails to improve mortality.

**Table 1**. Comparison of 28-day mortality of patients with SARS-CoV-2 treated with remdesivir, dexamethasone, hydroxycholoroquine, lopinavir, and interferon with/without O2 from the SOLIDARITY[39], ACTT-1[35], and RECOVERY[40] trials.

| Drug | 28-day mortality | No O2 | Low/hi-O2 | Ventilation |
|---|---|---|---|---|
| Remdesivir* (N=2743) | 301/2743 (12.5%) | 11/661 (2.0%) | 192/1828 (12.2%) | 98/254 (43.0%) |
| Control (N=2708) | 303/2708 (12.7%) | 13/664 (2.1%) | 219/1811 (13.8%) | 71/233 (37.8%) |
| Remdesivir** (N=541) | 59/541 (10.9%) | 3/75 (4.1%) | 28/327 (8.6%) | 28/131 (21.9%) |
| Placebo (N=521) | 77/521 (14.8%) | 3/63 (4.8%) | 45/301 (15.0%) | 29/154 (19.3%) |
| Dexamethasone*** (N=2104) | 482/2104 (22.9%) | 89/501 (17.8%) | 298/1279 (23.3%) | 95/324 (29.3%) |
| Usual care (N=4321) | 1110/4321 (25.7%) | 145/1034 (14.0%) | 682/2604 (26.2%) | 283/683 (41.4%) |
| Hydroxychloroquine**** | 104/947 (10.2%) | 69/862 (7.4%) | 35/85 (39.2%) | |
| Control | 84/906 (8.9%) | 57/824 (6.6%) | 27/82 (32.3%) | |
| Lopinavir***** | 148/1399 (9.7%) | 113/1287 (8.1%) | 35/112 (28.1%) | |
| Control | 146/1372 (10.3%) | 111/1258 (8.7%) | 35/114 (28.7%) | |
| Interferon-ß1a ****** | 243/2050 (12.9%) | 188/1911 (10.9%) | 55/139 (42.4%) | |
| Control | 216/2050 (11.0%) | 176/1920 (9.5%) | 40/130 (33.8%) | |

Remdesivir* - SOLIDARITY trial. Day 0: 200 mg; Day: 1-9: 100 mg i.v.;

Remdesivir** - ACTT. Day 1: 200 mg Day 2-10: 100 mg compared to placebo;

Dexamethasone *** - RECOVERY 6 mg oral/i.v. for up to 10 days;

Hydroxychloroquine **** - SOLIDARITY trial. Hydroxychloroquine sulphate a 200mg tbl at Hour 0, four tablets; Hour 6, four tablets; Hour 12, begin two tablets twice daily for 10 days;

Lopinavir ***** - SOLIDARITY trial. Lopinavir a 200 mg+ ritonavir 50 mg 2x 2 tablets for 14 days;

Interferon ****** - SOLIDARITY trial. Three doses over six days of 44 µg subcutaneous Interferon-ß1a.

***Dexamethasone***

According to the RECOVERY trial results, in the *dexamethasone* group, the incidence of death was lower than in the usual care group among patients receiving invasive mechanical ventilation (29.3% vs. 41.4%) and among those receiving $O_2$ without invasive mechanical ventilation (23.3% vs. 26.2%) but not among those who were not receiving respiratory support at randomization (17.8% vs. 14.0%) [40]. Based on these results, 6 mg of *dexamethasone* is recommended once daily for up to 10 days in COVID-19 patients on mechanical ventilation or who require supplemental $O_2$ but who are not on mechanical ventilation [38, 41].

***Chloroquine or hydroxychloroquine, lopinavir-ritonavir***

Although *chloroquine* or *hydroxychloroquine* were one of the medications which appeared to show great potential at the beginning of COVID-19 pandemic, their use has been stopped due to lack of efficacy. Numerous companies donated these medications for treating COVID-19 patients,however, the FDA revoked the emergency use authorization for this drug. Furthermore, the combined use of *hydroxychloroquine* and *azithromycin* is not recommended because of the potential adverse reactions. *Lopinavir/ritonavir* also did not demonstrate benefit in patients with COVID-19. As reported in Table 1, the interim WHO SOLIDARITY trial results indicate that *remdesivir*, *hydroxychloroquine*, *lopinavir* and *interferon* treatments had little or no effect on hospitalized COVID-19 patients, as indicated by overall mortality, initiation of ventilation and duration of hospital stay [39].

***Immunomodulatory medications***

Several medications used in modulating the immune response, such as interleukin-1 (*anakinra*) or interleukin-6 (*sarilumab, siltuximab, tocilizumab*) inhibitors are being used off-label and are being investigated. These medications have been proposed to suppress the cytokine storm [42].

***Convalescent plasma***

The convalescent plasma containing antibodies against SARS-CoV-2 virus collected from recovered COVID-19 patients is also being widely investigated. A randomised clinical trial with convalescent plasma therapy did not show any statistically significant improvement in clinical status or death rate [43]. However, this trial provided valuable information on the potential benefits of convalescent plasma, which may be useful in combination with antiviral drugs. According to some preliminary research, early administration of high dose intravenous immunoglobulin therapy may improve the prognosis of critically ill patients [44]. On August 23, 2020 FDA issued an emergency use authorization for convalescent plasma for the treatment of COVID-19 in hospitalized patients [45].

## Markers of disease evolution: what is available, what is needed

As the world faces the COVID-19 pandemic, markers enabling to predict the development of severe symptoms after SARS-CoV-2 infection are highly needed. Presence of cardiovascular risk factors (particularly arterial hypertension, diabetes mellitus and aging) and previous cardiovascular diseases reportedly expose to an unfavourable progression of COVID-19 [46]. As such, they can already provide an initial and rudimental model to risk stratify patients.

Mortality rate after COVID-19 is associated with elevation in the “classic” cardiac damage biomarkers, such as troponin T (TnT) and/or BNP/NT-proBNP [3, 47]. In line with that, COVID-19 patients who do not have significantly increased TnT levels show a lower mortality compared to patients without cardiovascular disease [5, 48]. This suggests that TnT and BNP/NT-proBNP concentration should be closely followed in patients with COVID-19 both for diagnostic (cardiac involvement) and prognostic purposes. Elevations of D-Dimers have also been associated with poor outcome [49]. The addition of other biomarkers such as the inflammatory cytokine IL6 and lymphocyte count will be also helpful to determine the individual risk of a patient.

Omics-based approaches recently discovered interesting metabolites in plasma of patients with COVID-19. Using both targeted and untargeted tandem mass spectrometry to profile the plasma lipidome and metabolome of COVID-19 patients with various degrees of severity and healthy controls, a panel of 10 plasma metabolites was found to distinguish COVID-19 patients from healthy controls with an area under the receiver-operating characteristic curve (AUC) of 0.975 [50].

Biomarkers that might be useful in indicating progression from mild to severe multi-organ complication in COVID-19 patients are summarized in Tables 2 and 3, which have been inspired in part by two important review articles and meta-analyses [51, 52]. A myriad of recent publications have reported associations between classical and emerging biomarkers and COVID-19 prognosis. Yet, only meta-analyses enrolling more than 200 patients are included in Tables 2 and 3. Among inflammatory and cardiac injury markers, decreased number of white blood cells, lymphopenia and thrombocytopenia and increased CRP, D-dimers, procalcitonin (PCT), lactate dehydrogenase (LDH), aspartate aminotransferase (AST), alanine aminotransferase (ALT), IL-6, cardiac troponin and CK-MB are associated with poor outcomes of COVID-19 patients, indicating their potential to aid in risk stratification and prediction of severe and fatal outcomes (Tables 2 and 3).

**Table 2.** Laboratory markers associated with poor outcomes after SARS-CoV-2 infection.

| **Sample size** | **WBC** | | **Lymphocytes** | | **Platelets** | | **D-dimer** | | **CRP** | | **PCT** | | **IL-6** | | **AST** | | **ALT** | | **Ref.** |
|---|---|---|---|---|---|---|---|---|---|---|---|---|---|---|---|---|---|---|---|
| | ↑↓ | **OR [95% CI]** | ↑↓ | **OR [95% CI]** | ↑↓ | **OR [95% CI]** | ↑↓ | **OR [95% CI]** | ↑↓ | **OR [95% CI]** | ↑↓ | **OR [95% CI]** | ↑↓ | **OR [95% CI]** | ↑↓ | **OR [95% CI]** | ↑↓ | **OR [95% CI]** | |
| 3962 | - | - | - | - | - | - | - | - | ↑ | - | ↑ | - | ↑ | - | - | - | - | - | 53 |
| 10491 | - | - | ↓ | 3.33 [2.51-4.41] | ↓ | 2.36 [1.64-3.40] | ↑ | 3.39 [2.66-4.33] | ↑ | 4.37 [3.37-5.68] | ↑ | 6.33 [4.24-9.45] | - | - | ↑ | 2.75 [2.30-3.29] | ↑ | 1.7 [11.32-2.20] | 51 |
| 1955 | ↓ | - | ↓ | - | ↓ | - | ↑ | - | ↑ | - | ↑ | - | - | - | ↑ | - | ↑ | - | 54 |
| 4662 | ↓ | - | ↓ | 4.5 [3.3-6.0] | ↓ | - | ↑ | - | ↑ | 3.00 [2.1-4.4] | ↑ | - | ↑ | 53.1% [36.0/70.0%] | ↑ | - | ↑ | - | 55 |
| - | ↓ | 0.93 [0.46-1.86] | ↓ | 1.66 [1.26-2.20] | ↓ | 0.88 [0.26-2.95] | ↑ | 1.50 [0.89-2.56] | ↑ | 1.41 [1.17-1.70] | ↑ | 2.94 [2.09-4.15] | - | - | ↑ | 2.27 [1.76-2.94] | ↑ | 1.60 [1.34-1.90] | 56 |
| 6320 | ↓ | 1.75 [1.21-2.54] | ↓ | 0.30[0.19-0.47] | ↓ | 0.56 [0.42-0.74] | ↑ | 3.97[2.62-6.02] | ↑ | 6.36 [3.22-12.5] | ↑ | 4.76 [2.48-9.14] | ↑ | 2.10 [1.02-4.32] | - | - | - | - | 57 |
| - | ↓ | - | ↓ | - | ↓ | - | ↑ | - | ↑ | - | ↑ | - | ↑ | - | ↑ | - | ↑ | - | 58 |
| 91621 | ↓ | - | ↓ | - | ↓ | - | ↑ | - | ↑ | - | ↑ | - | ↑ | - | ↑ | - | ↑ | - | 59 |
| 3027 | ↓ | 0.30 [0.17-0.51] | - | - | - | - | ↑ | 43.24 [9.92-188-.49] | - | - | ↑ | 43.24 [9.92-188.49] | - | - | ↑ | 4.00 [2.46-6.52] | - | - | 60 |
| 51225 | ↓ | 2.75 [2.02-3.9] | ↓ | -0.6 (-2.55--1.38) | ↓ | -36.06 (-49.24;-22.77) | ↑ | 3.22 [2.84-3.61] | ↑ | 68.31 [53.11-83.50] | ↑ | 0.52 [0.42-0.62] | ↑ | 43.64 [30.92-56.35] | ↑ | 17.41 [13.99-20.83] | ↑ | 2.18 [0.09-4.28] | 61 |

| | | | | | | | | | | | | | | | | | | | |
|---|---|---|---|---|---|---|---|---|---|---|---|---|---|---|---|---|---|---|---|
| 4631 | - | - | - | - | - | - | - | - | - | - | - | - | ↑ | RR 0.54 [0.27-0.81] | - | - | - | - | 62 |
| 5626 | - | - | - | - | - | - | ↑ | 1.4 [-2.04-(-0.77)] | ↑ | 64.03 [-68.88-(-59.19)] | - | - | - | - | - | - | - | - | 63 |

Legend: ALT – alanine aminotransferase; AST – aspartate aminotransferase; CRP – C reactive protein; IL-6 – interleukin 6; MA – meta-analysis; OR – odds ratio; PCT – procalcitonin; WBC – white blood cells; ↑ - increased; ↓ - decreased. The hyphen means not studied. Poor outcomes include in-hospital admission, intensive care unit admission, oxygen saturation <90%, severe disease, utilisation of invasive mechanical ventilation, and mortality. Adapted from [51, 52].

**Table 3.** Cardiac injury biomarkers associated with poor outcomes in COVID-19 patients.

| **Sample size** | **LDH** | | **CK** | | **Creatinine** | | **Troponin I** | | **CK-MB** | | **Ref.** |
|---|---|---|---|---|---|---|---|---|---|---|---|
| | ↑↓ | **OR [95% CI][c]** | ↑↓ | **OR [95% CI][c]** | ↑↓ | **OR [95% CI][c]** | ↑↓ | **SMD [95%CI]** | ↑↓ | **SMD [95%CI]** | |
| **-** | ↑ | 6.7 [2.4-18.9] | - | - | - | - | ↑ | 0.71 [0.42;1.00] | ↑ | 0.68 [0.48;0.87] | 56 |
| **6320** | ↑ | 2.03 [1.42-2.90] | ↑ | | ↑ | | | | | | 51 |
| **491** | - | - | - | - | - | - | ↑ | - | - | - | 58 |
| **91621** | ↑ | - | ↑ | - | ↑ | - | ↑ | 16% [11-22] | - | - | 59 |
| **3027** | ↑ | - | ↑ | - | ↑ | - | ↑ | 43.24 [9.92-188.49] | - | - | 60 |
| **51225** | ↑ | 8.86 [2.72-28.89] | - | - | ↑ | 5.30 [2.19-12.83] | ↑ | 0.02 [0.02;0.02] | - | - | 61 |
| **4631** | ↑ | 180.26 [131.02-229.51] | - | - | ↑ | 21.72 [16.72-26.71] | ↑ | 0.74 [0.19-1.30] | - | - | 62 |
| **5626** | ↑ | RR 2.20 [1.55-31.12] | ↑ | RR 1.89 [1.50-2.61] | - | - | ↑ | -1.55 [-2.23;-0.88] | ↑ | -4.75 [13.31;3.82] | 63 |
| **341** | - | - | - | - | - | - | ↑ | 25.6 [6.8-44.5] | - | - | 64 |
| **3118** | - | - | - | - | - | - | ↑ | 21.15 [10.19-43.94] | - | - | 65 |
| **4189** | - | - | - | - | - | - | ↑ | 0.53 [0.30-0.75] | ↑ | 0.62 [0.28-0.97] | 66 |
| **982** | - | - | - | - | - | - | ↑ | HR 2.48 [1.50-4.11] | - | - | 67 |

Legend: CK-MB – creatinine kinase -MB; HR – hazard ratio; LDH – lactate dehydrogenase; OR – odds ratio; RR – risk ratio; SMD – standardized mean difference; ↑ - increased; ↓ - decreased. Poor outcomes include in-hospital admission, intensive care unit admission, oxygen saturation <90%, severe disease, utilisation of invasive mechanical ventilation, and mortality. The hyphen means not studied. Adapted from [51, 52].

The role of the cardiovascular expression/activity of the putative SARS-CoV-2 receptor ACE2 as well as of the use of renin-angiotensin-aldosterone system (RAAS) inhibitors in SARS-CoV-2 susceptibility and COVID-19 disease severity have been a matter of debate [68-71]. However, the clear recommendation is to continue the administering of RAAS inhibitors or blockers in SARS-CoV-2 positive patients with underlying cardiovascular disease. Elevated angiotensin II levels have been found to correlate with lung injury and viral load, suggesting that administration of angiotensin 1-7 and angiotensin 1-9 may help in restoration of normal functioning of renin-angiotensin system by antagonizing the effect of abnormally increased angiotensin II [72].

Circulating RNAs represent a rich source of biomarkers with clinical utility due to their biological relevance, dynamic regulation in response to onset and progression of disease, tissue-specificity, and accessibility for non-invasive analysis using biofluids (“liquid biopsies”). Especially for diseases with diverse symptoms and complications such as COVID19, RNA biomarkers could provide important decision support. RNAs have shown some potential as cardiovascular disease biomarkers and may help in predicting unexpected cardiovascular events in COVID-19 patients. Although some clinical trials on miRNAs in COVID-19 have been started or are even completed (9 trials registered in clinicaltrials.gov database as of Nov 2020), none of them have been specifically designed to identify (mi)RNA predictors of cardiovascular outcome of COVID-19 patients. Table 4 gathers the currently available studies reporting regulations of non-coding RNAs (ncRNAs) in patients infected with SARS-CoV-2. Predicted messenger RNA targets as well as their proposed role in COVID-19 are also included in this table.

**Table 4.** Potential ncRNA biomarkers of COVID-19.

| ncRNA | Sample size | Type of sample | Regulation | Number of predicted target genes | Experimentally validated target genes in any disease | Experimentally validated target genes in COVID-19 patient samples | Proposed role in COVID-19 | Reference |
|---|---|---|---|---|---|---|---|---|
| miR-16-2-3p | 14 | Blood | ↑ | 71 | FGFR2, PDPK1 | - | - | 73 |
| miR-6501-5p | | | | 88 | - | - | - | |
| miR-618 | | | | 11 | MTDH, TLR-4, ATP6V1E1, HAT1, MCTS1, TGF-β2 | TLR-4, HAT1, TGF-β2 | TLR4 regulates inflammation;<br>HAT1: mitochondrial function, cellular senescence, and telomere attrition;<br>TGF-β2 induces expression of furin in HBE cells | |
| miR-183-5p | | | ↓ | 220 | PTEN, PIK3CA | PTEN | Regulator of SARS-CoV-2 ACE2-TMPRSS2-Furin-DPP4 axis | |
| miR-627-5p | | | | 25 | CDK6, SOX-2, LINC00958, lnc-UCA1 | - | - | |
| miR-144-3p | | | | 80 | PTEN, APP, FoxO1 | FOXO1<br>PTEN | FOXO1 regulates cell death downstream of several signaling pathways including CDK1, PKB/AKT1 and STK4/MST1<br>PTEN signalling is increased after SARS-CoV-2 infection | |

| | | | | | | | | |
|---|---|---|---|---|---|---|---|---|
| lncRNA DANCR | 563 | Lung tissue and blood | ↓ | - | miR-496/mTOR axis; miR-335-5p/miR-1972 and ROCK1 axis | mTOR | Regulator of Akt/mTOR/HIF-1 signaling pathway | 74 |
| lncRNA NEAT1 | | | ↑ | - | miR-129-5p/KLK7 axis; | RUNX3, SPI1 | RUNX3 regulates DANCR and is related to inflammatory reaction in the lung;<br>SPI1 controls DANCR expression in the brain and in epithelial cells | |
| miR-21-5p | | | ↑ | 139 | TGFBI, MAPK1 | DANCR, NEAT1 | DANCR and NEAT1 can block inflammation via interacting with other ncRNAs, sponging miRNAs, or affecting TFs (e.g. STAT3) | |
| miR-22-3p | | | | 162 | WRNIP1, HMGB1 | HMGB1 | Exogenous HMGB1 induces the expression of SARS-CoV-2 entry receptor ACE2 | |
| miR-335-5p | | | | 63 | Rb1, CARF, SGK3 | - | - | |
| miR -19a-3p | | | | 241 | UBAP2L, PSG10P, IL1RAP | - | - | |
| miR-1207-5p | 18 | Lung tissue | ↑ | 147 | CSF1 | CSF1 | Enhances macrophage recruitment and activation and its overexpression may contribute to acute inflammation | 75 |

| | | | | | | | | |
|---|---|---|---|---|---|---|---|---|
| miR-21-5p | Discovery: 33 Validation: 65 | Serum | ↑ | 41 | RASGRP1, BCL2, SMARCA4, SPRY2, DUSP10, TIMP3, SOX5, MTAP, RECK, PIAS3, TGFBR2, PTEN, E2F1, LRRFIP1, TPM1, NFIB, APAF1, BTG2, PDCD4, RHOB, ANP32A, SERPINB5, BMPR2, DAXX, TP63, MSH2, MSH6, ISCU, EIF4A2, ANKRD46, CDK2AP1, PPARA, FASLG, SMAD7, SERPINI1, DDAH1, HPGD, MYD88, IRAK1, VHL, GDF5, IL12A, CASC2, DNM1L | TIMP3<br>PTEN | SARS-CoV-2 reduces TIMP3 mRNA expression in alveolar epithelial cells, that likely promotes greater ADAM17 activity in COVID-19 patients.<br><br>PTEN signalling is increased after SARS-CoV-2 infection | 76 |
| miR-155-5p | | | ↑ | 70 | MEIS1, TAB2, MECP2, SOCS1, MLH1, INPP5D, SMAD5, HIVEP2, ZNF652, | TAB2<br>SOCS1<br>TP53INP1<br>FADD | TAB2 is associated with vascular inflammation<br><br>SOCS1 is a key checkpoint regulator of the immune system | |

| | | | | | | | | |
|---|---|---|---|---|---|---|---|---|
| | | | | | BACH1, APC, SMAD1, SDCBP, MYO10, CLDN1, CEBPB, RHOA, AGTR1, RNF123, TP53INP1, IKBKE, KDM3A, SPI1, FOXO3, RUNX2, JUN, ETS1, CYR61, SMAD2, MYB, SKI, CKAP5, SOX6, CSF1R, FADD, NOS3, MYLK, PSIP1, ANXA2, HBP1, NFKB1, E2F2, PIK3R1, MMP16, MYC, SEL1L, DOCK1, RAD51, MXI1 | | TP53INP1 induced cell death by an autophagy- and caspase-dependent mechanism<br><br>The FADD/caspase-8 axis regulates TNF-α and IFN-γ co-treatment-induced inflammatory cell death independent of intrinsic apoptosis in macrophages | |
| miR-208a-3p | | | ↑ | 3 | CDKN1A, MED13, ETS1 | - | | |
| miR-499-5p | | | ↑ | 43 | FOXO4, PDCD4, ETS1 | FOXO4 | Down-regulated upon SARS-CoV-2 infection, associated with cellular signaling | |

Legend: CSF1 - colony stimulating factor 1; DANCR - anti-differentiation lncRNA; FADD – Fas associated via death domain; FOXO4 - forkhead box O4; FOXO1 - forkhead box O1; HAT1 - Histone acetyltransferase 1; HMGB1 - high-mobility group protein 1; lncRNA – long noncoding RNA; mTOR - Mechanistic Target Of Rapamycin Kinase; ncRNAs – non-coding RNAs; NEAT1 - nuclear paraspeckle assembly transcript 1; PTEN - Phosphatase And Tensin Homolog; RUNX3 - RUNX family transcription factor 3; SOCS1 – suppressor of cytokine signaling 1; SPI1 - Spi-1 proto-oncogene; TAB2 – TGF-beta activated kinase 1 (MAP3K7) binding protein 2; TGF-β2 - Transforming Growth Factor Beta 2; TIMP3 - TIMP metallopeptidase inhibitor 3; *TLR*-4 - Toll Like Receptor 4*;* TP53INP1 – tumor protein p53 inducible nuclear protein 1;

↑ - upregulated;↓ - downregulated. Predicted miRNA-target interactions were performed using miRWalk 3.0, miRDB 6.0 and miRTarBase 8.0 databases. Experimentally validated target genes in any disease (mostly cancer) were obtained from miRTarBase 8.0. Experimentally validated target genes in COVID 19 and their proposed roles were obtained through literature search. The authors apologize for the many references that could not be added to this table due to space restrictions.

Given the disproportionate impact of COVID-19 in ethnic minorities, it is essential to clarify if biomarkers are of use in such populations and if so how they could be ad-hoc adapted. Not only cardiac but also endothelial biomarkers deserve attention [77]. Gender-medicine considerations for COVID-19 cardiovascular risk stratification are also of paramount importance. Women appear to be better protected, as men display higher mortality rates (ranging from 60 to 75 %) [78]. Should this be due to a protective effect of oestrogens, perimenopausal and postmenopausal women without hormonal replacement therapy could be considered at higher risk of cardiovascular death following COVID-19. Preclinical evidence suggests that sex may influence the expression of the ACE2 receptor [78]. Hence, the examination of sex differences should be an integral part of COVID-19 directed research projects. This is especially crucial as sex-specific RNA biomarkers may help in tailoring future healthcare.

## Networking and coordination efforts for multinational multi-center studies on cardiovascular RNA markers

For a global pandemic of this kind, worldwide efforts are needed to understand the infectious agent, to develop diagnostic tools, treatments and also to monitor the well-being of those infected with SARS-CoV-2 in the following years. For robust development of biomarkers or treatments, their effectivity must be validated in numerous cohorts, internationally in different demographics and on a large scale.

Addressing the increasing challenges posed by communicable diseases thus calls for multidisciplinary and multi-centre international cooperation to link available data, tools and expertise, which will otherwise only be sub-optimally exploited at regional or national levels. A truly integrated approach coordinating and facilitating the access to and sharing of biological resources, data, advanced technological facilities and expertise, within a common research roadmap, is needed to exploit the full potential of the various resources. As COVID-19 incidence and clinical outcomes have been shown to be greatly influenced by many biological and environmental factors, the need to integrate data across the various settings worldwide is critical to increase the precision of analyses and to deliver meaningful results.

Through the EU-CardioRNA COST Action [79], in April 2020, a call was placed to assemble a taskforce of clinicians and translational scientists working with COVID-19 patients to join forces in an international effort. This was communicated internally within the Action network as well as externally on the Action website and professional (social) media (https://cardiorna.eu/news/cost-actions-unite-efforts-in-the-fight-against-covid-19/) [80]. In total, 38 institutions and 22 countries responded to the call. Members of the taskforce have access to COVID-19 patient clinical data, blood samples and other biospecimen and/or expertise in analysis of biomarkers in liquid biopsies.

With the clear elevated risk of COVID-19 in aged individuals and patients with cardiovascular disease, the task force aims to monitor (re-) hospitalization rates, mortality rates from cardiovascular disease in those with exposure to SARS-CoV-2, as well as to identify RNA biomarkers reflecting cardiovascular health [81]. We encourage medical professionals in the taskforce to perform functional follow-up of COVID-19 infection including echocardiography or cardiovascular magnetic resonance imaging, where possible. Using the numerous and extensive patient databases across multiple centres involved, epidemiological analyses and clinical statistics will be performed to identify differential risk of COVID-19 infection and response as well as cardiovascular effects according to comorbidities and patient characteristics. This is especially important to identify individuals most at risk in the event of long-term presence of the SARS-CoV-2 in the population, as well as to further understand the mechanism of infection and morbidity.

Regulatory RNAs are emerging as stable reliable circulating molecular indicators of cardiovascular health [82]. With expertise in regulatory RNA biomarkers detectable in peripheral blood samples, the EU-CardioRNA taskforce will analyse these RNAs and overlay with clinical information in search of biomarkers for cardiovascular outcomes of COVID-19 infection. Such a study and the translation of the results into clinical application would not be feasible without the integration of complementary expertise and resources from the various actors (cardiology and infectious disease, biomarkers, RNA, cardiovascular research, clinical studies, bio banking, artificial intelligence, data management, biostatistics, bioinformatics, technology transfer, etc.).

## Technical challenges and requirements in the RNA-study

The quantitative analysis of RNAs in biological samples faces several technical challenges that must be overcome in order to generate robust and reproducible results. Specifically, the analysis of circulating RNAs is complicated by a variety of pre-analytical settings that impact the analysis as well as the analytical challenge to deal with very low RNA concentrations.

To date, whole blood, serum and plasma are the most widely explored liquid matrices for circulating RNA analysis. Analysis of whole blood can be biased by red blood cells and platelets, which are a rich source of small RNAs despite being anucleate [83]. Thus, protocols for specific depletion of certain types of RNAs have been developed for whole blood that improve sensitivity for other types of RNAs [84]. Serum and plasma as the liquid components of blood can behave quite differently due to the release of RNAs during platelet activation and blood coagulation after which serum is collected [85]. Therefore, results for RNA biomarker analysis are oftentimes not comparable between serum and plasma [86]. In addition, contamination of serum or plasma with cellular RNA derived from red blood cells due to haemolysis [87, 88], or platelets due to variable pre-analytical processing [89], can confound the analysis and lead to false-positive or false-negative results [90].

Currently, only few studies have attempted to address sources of bias for other types of liquid biopsies. For example, in case of urine it is known that donor-dependent differences in volume based on hydration status result in highly variable RNA concentrations that require normalization prior to analysis using for example urinary creatinine levels [91].

In biofluids, RNAs are associated with two main types of RNA carriers, which facilitate transport and protect their RNA cargo from degradation: protein complexes and extracellular vesicles (EVs). At least in terms of small RNAs, it is known that the majority of extracellular RNAs in plasma or conditioned media is associated with protein complexes[92, 93]. This means that total RNA isolation and analysis from these matrices mainly reflects the protein-associated RNA fraction, and that the separate analysis of RNAs that are selectively released via EVs can reveal different results [94]. It is important to note that RNA analysis in EVs is anything but trivial and requires careful optimization of EV isolation and characterization and reporting according to the MISEV standard developed by the International Society of Extracellular Vesicles [95].

The analysis of RNA integrity and abundance obtained by RNA isolation is hampered by low concentrations. Thus, either highly sensitive methods using RNA specific dyes should be used and internal process controls such as spike-in oligonucleotides ("spike-ins") can be useful to monitor RNA recovery and analytical variability and to normalize RNA expression data in biofluids in the absence of robust endogenous RNA references.

Analytical methods for circulating RNA quantification must also be highly sensitive to cope with low concentrations. Reverse-transcription quantitative PCR (RT-qPCR) is a gold-standard technology for this purpose. However, low throughput and high cost for using RT-qPCR in genome-wide RNA biomarker discovery have restricted its use to targeted analyses for biomarker validation. This limitation resulted in the uptake of next-generation sequencing (NGS) for untargeted RNA biomarker discovery. Since early on it was observed that the abundance and stability of small RNAs in biofluids was surprisingly high, small RNA sequencing was rapidly adopted for biomarker identification in liquid biopsies [96].

The challenges for using small RNA NGS for circulating RNA analysis are 1) the extended PCR pre-amplification that is need to obtain sufficient input material but is potentially resulting in PCR duplicates, 2) adapter-ligation bias leading to over- and under-representation of certain RNAs in the library, and 3) the relative quantification that restricts the main use to cross-sectional comparisons between selected groups. To overcome these challenges, unique molecular indices can be included in the adapter sequences to identify and remove PCR duplicates prior to data analysis [97]. Second, sophisticated adapter-design such as randomized ends or single ligation protocols have been shown to reduce the ligation-bias and reduce adapter-dimers [98, 99].. Finally, the addition of spike-in calibrators with randomized ends and optimized concentration ranges can be used to normalize small RNA NGS data and achieve absolute quantification that is less sensitive towards changes in the (small) RNA composition of a sample [100].

Recently, also the application of total RNA sequencing for RNA biomarkers discovery in liquid biopsies has advanced to explore the full spectrum of RNAs. A stranded total RNA sequencing kit appeared to be sufficiently robust, accurate and precise to quantify thousands of genes in platelet-rich and platelet-free plasma, urine, and conditioned medium as well as EVs isolated from these matrices [101]. EVs from platelet-free plasma showed a large percentage (>80%) of short reads that were too short to be aligned. This was not observed for total RNA from platelet-free plasma and platelet-rich plasma, and total RNA as well as EV-RNA from urine and conditioned medium. This might suggest that RNA released from cells via EVs into the blood stream might be fragmented endogenously. In terms of gene-biotypes, protein coding genes made up the majority (>70%) of reads for all matrices except platelet-rich plasma, followed by pseudogenes, long noncoding RNAs (lncRNAs), and miscellaneous RNAs [101].

Overall, the planning of ideal RNA biomarker study should in the first step consciously decide which biological matrix and RNA carrier are most relevant and practical, secondly, implement standardized protocols for sample collection and sample quality control at the study sites, and thirdly, take advantage of a well-characterized, fit-for-purpose validated, NGS protocol for genome-wide total RNA and small RNA quantification in low RNA input samples.

## Data handling and integration

Data infrastructure that curates, integrates and analyses clinical and experimental data from several COVID-19 cohorts is pivotal to make harmonized data available to research network members in order to unravel cardiovascular RNA markers of SARS-CoV-2 infection. Systematic collection, and application of standards play an important role in managing and handling cohort data and its meta-data efficiently. They facilitate FAIR (Findable, Accessible, Interoperable and Reusable) use of the data, which provides a solid foundation for systematically discovering, retrieving, understanding, integrating, disseminating, exchanging, reusing the data and reproducing research results and outcome.

***Making data findable, including provisions for metadata***

In order to make the data discoverable, the following rules should be ensured:

- Data sets need to be assigned a unique identifier within the project. The data management team ensures that the identifier is globally unique.
- Accompanying metadata such as the study protocol, experimental parameters etc. should be provided. This will make it possible for members of research networks to fully grasp the experimental setup and data content.

- Project should follow standards defined for the different data domains, e.g. the clinical data interchange standards consortium (CDISC) standards for collecting clinical and non-clinical data, or the minimum information about a microarray experiment (MIAME) for microarray experiments. An extensive list of recommended standards is defined in the eTRIKS - Standards Starter Pack Standards Guideline [102].
- The metadata of datasets should be collected by using the templates developed by ongoing efforts such as IMI-FAIRplus, COVID-19 Research Data Alliance working groups. The metadata should be published in a searchable registry or data-catalogue (e.g. IMI FAIRplus catalogue [103]) to enable findability of datasets.

***Making data accessible***

Data should be made available to broader audience in accordance with the access model that will be defined by participant informed consent, and ethics/institutional review board approvals. This should include descriptions and data formats and in compliance with legal obligations, in particular the General Data Protection Regulation (GDPR). Data security is of paramount importance for protection of personally identifiable information.

***Making data interoperable***

Harmonization of data and metadata by applying standard ontologies, controlled terminologies, and state-of-the-art data models is pivotal for interoperability of the data that will facilitate cross-study analysis. Clinical and phenotype data should be standardised by using state-of-the-art standards such as the CDISC standards: Study Data Tabulation Model (SDTM), Clinical Data Acquisition Standards Harmonization (CDASH), and Analysis Data Model (ADaM). All clinical datasets from various cohorts should be mapped to International Severe Acute Respiratory and emerging Infection Consortium (ISARIC) COVID-19 eCRF [104]. In addition, application of controlled terminologies and ontologies described in the eTRIKS Standards Starter Pack including ICD-11, MedDRA, WTO ATC codes, Human Phenotype Ontology (HPO), LOINC etc., is important to standardise and harmonise contents/values of clinical variables.

Omics or molecular data and associated metadata from both single-cell and bulk samples should be standardised using ISA (Investigation, Study and Assay) framework [105] and corresponding minimum information guidelines (MIGs) such as MIAME format for transcriptome data, MIGS-MIMS (minimum information about a genome/metagenome sequence), and MINSEQE (minimum information about a high-throughput NucLeo Pharmatide sequencing experiment) for both genome and transcriptome data. Study data tabulation model implementation guide (SDTMIG) pharmacogenomics/genetics (PGx) standards are useful to represent the genetic biomarkers including genetic variation, genotyping and RNA expression data. To represent molecular entities within omics data, it is important to use identifiers from standard databases such as ENSEMBL gene, NCBI gene, ENSEMBL transcript for messenger RNA, UniProt for proteins, NCDB dbSNP for SNPs, GO for gene ontology and KEGG (Kyoto Encyclopedia of Genes and Genomes)/Ingenuity for pathways. Molecular data, for example, the transcriptome of the bio-samples and each transcript are mapped to ENSEMBL transcript identifier (one of the stable and persistent identifiers). The corresponding genes, proteins are mapped to EMSEMBL genes, NCBI genes, UniProt identifiers and involved biological processes, cellular components, molecular functions using GO and KEGG identifiers. These stable identifiers provide cross-references to other biological databases and thus facilitate the interoperability of the molecular (-OMICS) data.

In addition to applying the state-of-the-art standards for clinical and omics data, application of standards in data management to guarantee data security, data privacy and compliance with GDPR and ethical guidelines are necessary. Given the sensitive nature of human data, the data and computing environment must be access controlled and in/output data flows should be encrypted, site restricted and equipped with two-factor authentication wherever needed.

***Increase data re-use (through clarifying licences)***

The long-term sustainability for the database, analysis portal and related outputs (results, tools, software modules and algorithms) should be planned in advance. For archiving, preservation and long-term usage of the data and software tools/algorithms, research network partners should have the capacity to provide long-term sustainability of translational research data through GDPR-compliant hosting and tools. The process should follow well-defined access criteria and data protection needs. We recommend to prepare a sustainability plan for defining the rules to fulfil the legal processes (including addressing the issue of institutional data access committee responsibility), governance and the economic viability of the database.

### *Data integration*

A robust and secure data management and analysis platform, for example through a software portal and database, is important for the collection and integration of harmonized clinical, healthcare (electronic health records) data and pre-processed omics (molecular) data, imaging data, and real-world sensor/mobile data, biobank sample data and metadata from various COVID-19 projects (Figure 2).

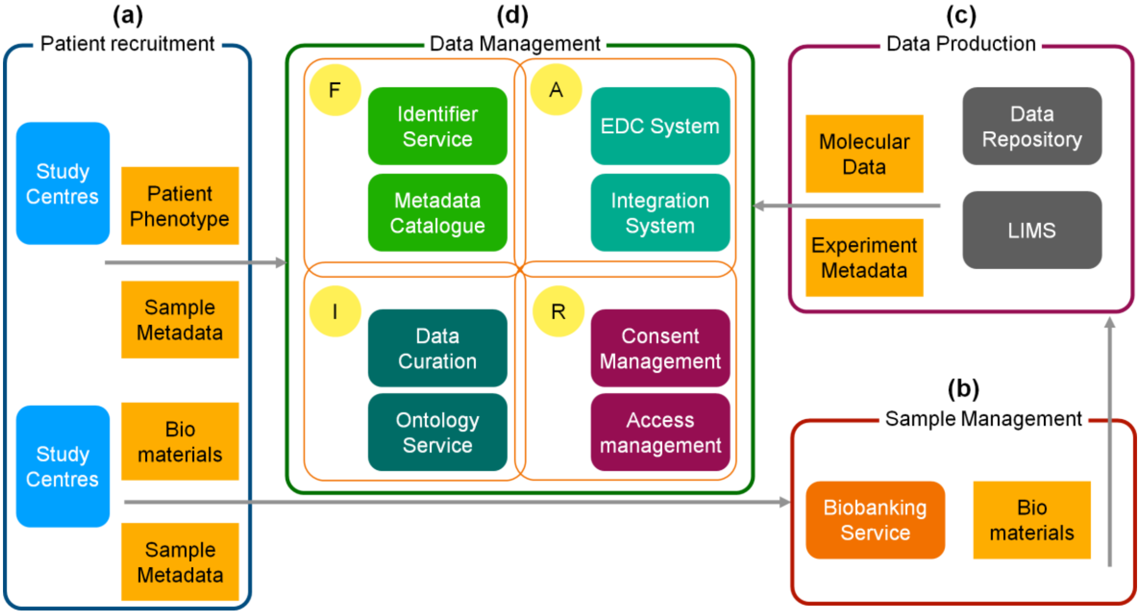

**Figure 2.** A reference setup of data platform to support FAIR (Findable, Accessible, Interoperable, Reusable) data management. (a) Patient Recruitment sites will collect patient phenotype data (including clinical data defined in the defined in case report form) and sample metadata for the bio-materials collected. The phenotype data and sample metadata will be collected using the Electronic Data Capture (EDC) system hosted at the Data Management site. Bio samples will be transferred to (b) the Sample Management Site that handles the Biobanking service and provides treated samples to (c) the Data Production site. There, the molecular data will be measured. Metadata about the experiments as well as the molecular data will be managed first in the Lab Information Management System (LIMS) and further transferred to the (d) Data Management site. The Data Management site will be equipped with Identifier Service and Metadata Catalogue for data Findability (F), EDC system and data Integration System for data Accessibility (A), Data Curation platform and Ontology Service for data Interoperability (I) and Consent Management system as well as Access Management system for data Reusability (R).

.

Such a data portal should also provide secure, easy and robust interface for the input and integration of new data from ongoing recruitment of cohort studies. Analytical tools from existing initiatives/packages such as I2B2 [106], tranSMART [107], SmartR [108], EGA (European Genome-phenome Archive) [109], and eTRIKS platform [110] are very useful to perform integrated data analysis and hypothesis generation. In order to store, process and analyse imaging data, for example chest X-ray images from COVID-19 patients, a dedicated open source imaging informatics solution such as XNAT [111] should be integrated into the platform instead of only storing the images in a file system. Such a portal will enable researchers to perform cross-study comparisons, slice and dice the cohorts based on certain clinical features and run built-in workflows from the graphical user interface. An application programming interface to enable batch/programmatic interaction with the portal will provide structured and harmonized data to bioinformaticians, statisticians and data scientists working with large amounts of data.

## Data analysis, biostatistics, artificial intelligence

After the data on RNA and clinical data are collected, secured, pre-processed and integrated, most informative biomarkers to predict major adverse cardiovascular events (MACE) and mortality of COVID-19 patients shall be identified. This identification can rely on biostatistical and machine-learning (ML) methods. Afterwards, ML should be utilized to build a classifier to predict MACE and mortality based on these biomarkers. For this approach to be used, RNA expression data accompanied by demographic and clinical data of patients are required, as well as information on MACE and mortality. To our knowledge, such data is not yet available – or has not yet been compiled from different patient cohorts – in a sufficient number of patients, allowing for application of ML methods. The dataset – once available – needs to be properly organized for analysis. It should be split into training, validation and test datasets. The training dataset is intended for biomarker discovery and model training, the validation dataset allows model selection and hyperparameter optimization, and the test dataset is for final testing. If the available dataset does not contain data from a large number of patients, k-fold cross validation may be used to split the data. If the distribution of classes (with vs. without MACE, or dead vs. alive) is imbalanced, resampling of the training data (either under-sampling the majority class or over-sampling the minority class) may be appropriate[112, 113].

### *Biomarker identification*

The most basic approach to identify predictive RNAs is differential expression analysis: RNAs that are significantly over- or under-expressed in patients who experienced a MACE or died, compared to those who did not, are potential biomarkers. Various statistical methods can be used for this [114]. However, this approach is simplistic, mainly in that it does not take into account interactions between the RNAs, so it can only serve as the first step. Two more sophisticated approaches can be explored: Bayesian variable selection (BVS) and feature selection.

Bayesian variable selection is a state-of-the-art statistical approach for selecting informative predictors such as RNA biomarkers [115]. One first picks a class of models, such as linear or logistic regression models, to predict the end-point of interest (e.g. MACE or mortality) based on the predictors (RNA quantities). The goal is to select from this class of models those able to accurately predict end-points. To do so, prior probability distributions of their parameters need to be set first. The most appropriate strategies to do this is subject of ongoing research, but one of accepted automatic methods can certainly be used. We believe, though, that information on RNA's biological function from the NONCODE database [116], or overlap with genomic loci related to cardiovascular disease, could yield more informative biomarkers. Based on the models' prior probabilities and the collected data, one computes their posterior probability using the Bayes rule, where good models are the ones with a high posterior probability. Since the space of models is too large to search exhaustively, Monte-Carlo sampling is used, which can relatively quickly identify accurate models.

Feature selection is an approach that selects informative features (RNA biomarkers) to be used to train ML models that predict the end-point of interest (MACE or mortality) [117]. There are three main groups of feature-selection methods. Filter methods consider each feature in isolation and are similar to differential expression analysis, so they are rarely the best option. Embedded methods are a part of some ML algorithms. Their quality depends on the quality of the algorithm they are derived from, but they can take into account some interactions between features. Wrapper methods are the most complex ones and are conceptually similar to BVS. They search the space of feature combinations, and evaluate each combination by training a model on it and checking the model's accuracy. Since the space of feature combinations is again too large to search exhaustively, various types of greedy search are typically used. The main advantage of simple approaches, such as differential expression analysis or filter feature selection, is the clear justification for the selection of each biomarker. The disadvantage is that they can provide redundant biomarkers or fail to identify RNAs having biomarker potential only when combined with others. The advantage of BVS and more advanced feature selection is that they provide sets of biomarkers that perform well in combination. The disadvantages are that they are somewhat opaque and computationally expensive. Wrapper methods appear to be the most flexible and potentially most powerful methods to identify predictive biomarkers.

From the two approaches, we recommend Bayesian variable selection and feature selection, either the one that results in better risk-prediction models on the validation dataset, or the combination of both can be used. They can be combined in sequence (one making the first selection and the other refining it) or in parallel (by using the intersection or union of the biomarkers selected by the two approaches). The best approach depends on the dataset and the outcome to predict, and needs to be determined experimentally.

***Cardiovascular/COVID-19 risk prediction***

After identifying the most informative RNA biomarkers, these – together with phenotype (demographic and clinical) data – are fed into ML algorithms to build risk-prediction models.

Figure 3 depicts the workflow of biomarker identification and COVID-19 risk prediction. Details of data collection and data management are depicted in Figure 2. The workflow starts with the data collection and management. This is followed by biomarker identification using the training dataset (cf. section Biomarker identification) and machine learning model development with the validation dataset. Note that even though biomarker identification can be done independently of phenotype and clinical data, such data is often included in the prediction models. This enables one to analyse their capacity to predict MACE and mortality alongside with the RNA biomarkers. Finally, the prediction model is thoroughly evaluated using the test dataset.

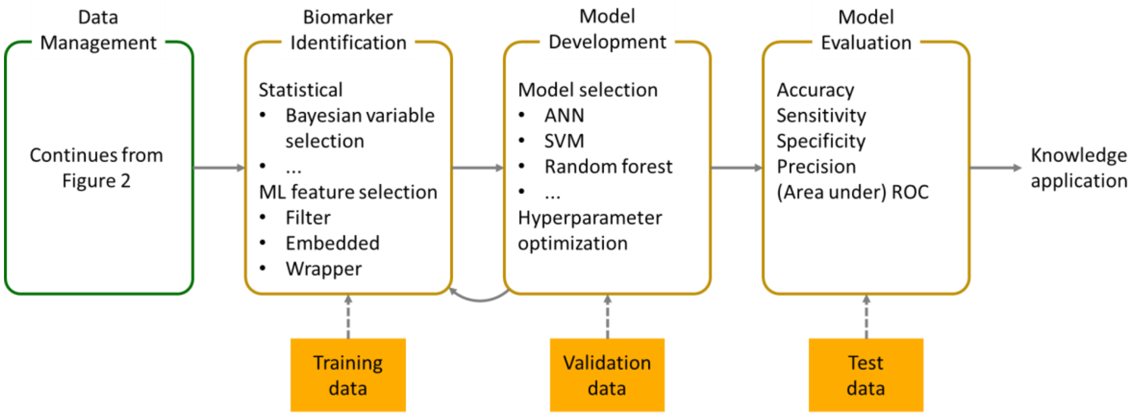

**Figure 3**. Biomarker identification and COVID-19 risk prediction workflow.

The most common ML algorithms that have been successfully applied to problems that use omics and clinical data include artificial neural networks (ANNs), support vector machines (SVMs) and ensemble methods such as random forest.

ANNs have been designed to mimic human neural architecture. ANNs are able to effectively capture complex non-linear relationships in the data and are thus suitable for complex RNA data combined with clinical data. However, they are often computationally demanding, and compared to other algorithms they have many parameters that require tuning in order to optimize the prediction accuracy. Deep learning models are ANNs with multiple hidden layers. Many different deep neural network architectures exist [118]. Figure 4 depicts an example of an ANN, with input data using the phenotype (demographic and clinical) and molecular data (RNA biomarkers). ANN consists of interconnected neurons, arranged in input layer, one or more hidden layers and an output layer. During training of ANN, the model learns from the examples provided in the training set.

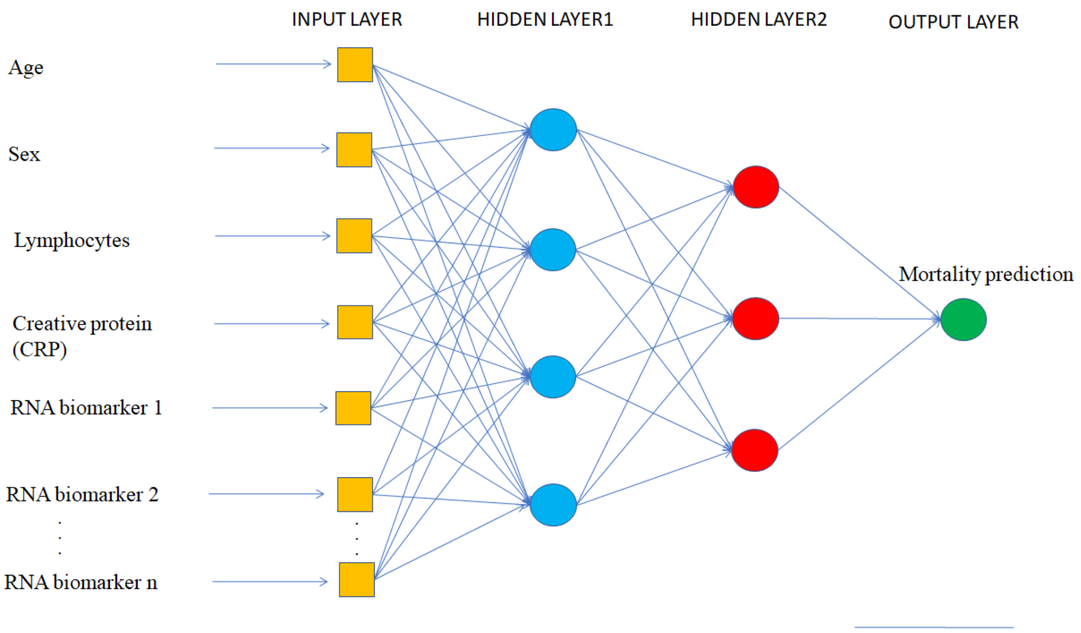

**Figure 4**. Architecture of an ANN. Input layer contains the collected patient phenotype (demographic and clinical) and molecular data (RNA biomarkers), followed by two input layers, and an output layer which in this case predicts mortality, but could also predict MACE or other clinical outcomes.

SVM is another ML algorithm that is able to capture data non-linearity. SVM applies a kernel to map data into multidimensional space. A SVM model is a hyperplane that splits the classes in this multidimensional space in a way that minimizes the prediction error during data classification. The selection of the kernel function is crucial to the algorithm's performance. Compared to ANNs, SVMs tend to be more resistant to overfitting (better handle noise in the training data) and require less memory.

Ensemble methods are a popular approach that has been successfully applied to high-dimensional biomedical datasets with small sample size. The idea behind ensemble methods is to combine several base classifiers that will produce better classification results than a single classifier. One of the most successful ensemble methods is random forest. Random forest uses a set of decision trees that form a forest. In order to avoid overfitting, each decision tree in the forest uses a random subset of samples from the training set, and a random subset of features. Classification is then performed based on the majority vote of the trees. For example, the FEELnc tool uses random forest for annotation of lncRNAs and achieves an AUC of 0.97 [119]. The lncLocator tool uses an ensemble of support vector machine and random forest classifiers to predict lncRNA subcellular localization [120].

Considering the recent success of deep learning, we believe this method to be worth investigating. Due to the many parameters in deep ANNs, it typically requires more data than other methods, hence it may become unsuitable for datasets limited in size. In this case, ensemble methods are likely to provide better results. To evaluate the performance of the classification methods, various measures such as classification accuracy, sensitivity, specificity and precision can be used [121]. The AUC is a particularly suitable performance indicator, since it evaluates the performance of models over all possible trade-offs between type 1 and 2 errors.

## Translational aspects: development of diagnostics and therapy for COVID-19.

The rapid spread, high mortality in some geographical areas, and the yet largely unknown long-term consequences of COVID-19 including cardiovascular pathologies all highlighted the need to develop effective diagnostic and prognostic biomarkers and therapeutics against SARS-CoV-2. Basic research and development of novel biomarkers and therapeutics run in parallel on the basis of broad collaboration between key players of the biomedical field including industrial and academic partners, national governments and regulatory agencies as well as investors. Ongoing repositioning of existing drugs as well as development of novel drugs, vaccines and a variety of medical devices for prevention and treatment of COVID-19 have been at the front line of very recent research activities.

Development of RNA diagnostics and therapeutics, especially small non-coding RNA compounds, attracted the attention of the pharmaceutical industry in the past few years that has been further accelerated by the rapid outbreak of COVID-19. Indeed, development of RNA molecules for diagnosis, prognosis, and treatment of SARS-CoV-2 and other RNA viruses has been recently proposed [122, 123]. Currently there are 9 ongoing or completed clinical trials when searching for miRNA and COVID-19 in the clinicaltrials.gov platform, showing the rapidly increasing activity of translational research in this field. Moreover, extracellular vesicles −as important players in the life cycle of RNA viruses as well as cargo particles for non-coding RNAs −may provide opportunities for more sensitive diagnosis and targeted therapies for SARS-CoV-2 [124, 125]. Although there are currently no examples of molecular diagnostic assays based on cardiovascular RNA biomarkers of COVID-19 and utilizing digital PCR as a means to quantify circulating RNA transcripts, we believe that this technology holds great promise and may rapidly be applied to COVID-19 tests.

Another aspect of the COVID-19 pandemic is the need for cardioprotective strategies to prevent the long term cardiovascular consequences of the disease. Yet, despite intensive efforts, the development of cardioprotective therapies has been unsuccessful in the last 3 decades [126]. Small non-coding RNAs fingerprints of COVID-19 itself and the different comorbidities and their co-medications that affect the infection may provide a useful tool to develop diagnostic and prognostic markers and to discover novel drug targets to prevent and treat COVID-19 and its cardiovascular consequences [127]. Understanding the molecular interactions between SARS-CoV-2 and its host as well as the influence of cardiovascular risk factors, comorbidities, and medications on clinical outcomes may significantly speed up the lengthy process of development of diagnostics and therapeutics not only against COVID-19 but also other diseases [127-129].

## Conclusion and perspectives

COVID-19 has brought about an unexpected and unprecedented historical period, worldwide. Despite the tremendous efforts and reactiveness of all stakeholders from the broad healthcare sector ─ clinicians, healthcare staff, researchers, funding bodies, and regulatory authorities ─, the burden of COVID-19 is enormous, both medically, socially as well as economically.

The research field has been very reactive and multiple networks of experts and task forces have been formed to tackle the challenge of finding drugs and biomarkers of COVID-19. Building an effective coordination of large interdisciplinary networks involved in multi-centre studies is key for success of biomarker projects. RNA biomarkers combined with artificial intelligence-based strategies will certainly help in building algorithms to aid in clinical decision making and personalization of healthcare through risk stratification of patients. Efficient academia-industry partnerships are essential to rapid marketing and clinical use of novel disease biomarkers. Novel tools based on systems biomedicine concepts and artificial intelligence methods are needed to speed up the translational process and clinical application.

While it is obvious that the cardiovascular burden associated with SARS-CoV-2 infection is alarming and deserves great attention during healthcare of COVID-19 patients, it is also important to keep in mind that more than a third of hospitalized COVID-19 patients present psychological distress and neurological manifestations such as headache, ischemic stroke, seizures, and other diverse encephalopathies [130]. SARS-CoV-2 has been detected in the brain and cerebrospinal fluid [131], and is associated with encephalitis. Various neurological sequelae have been associated with the Spanish influenza pandemic and other coronaviruses [132]. Therefore, a deeper knowledge of the host-pathogen interactions involving regulatory RNAs [82] in the brain-heart axis [133] may provide novel avenues for discovery of biomarkers and therapeutic pathways to improve healthcare and prepare for future pandemics.

**Conflicts of Interest**

L.B  is founder and shareholder of Glycardial Diagnosis SL; received a research grant from AstraZeneca; hold advisory board work for Sanofi, Bayer and AstraZeneca,; and received speaker fees from Lilly, MSD-Boehringer and AstraZeneca (all outside of this work).

L.D.W. is founder and shareholder of Mirabilis Therapeutics BV.

P.F. is the founder and CEO of Pharmahungary Group, a group of R&D companies.

M.H. is employed by TAmiRNA GmbH and company shareholder. M.H. holds patents related to diagnostic and therapeutic applications of microRNAs.

F.M. acted as consultant for Amicus Therapeutics  on a topic not related to the manuscript.

T.T. is founder and shareholder of Cardior Pharmaceuticals GmbH. T.T. received support (including speaker fees) and/or holds advisory board activities for Boehringer Ingelheim, Sanofi-Aventis, Takeda, Amicus Therpeutics, Novo Nordisk (all outside the field of this manuscript).

Y.D. holds patents related to diagnostic and therapeutic applications of RNAs.

**Acknowledgments**

This article is based upon work from EU-CardioRNA COST Action CA17129 (www.cardiorna.eu) supported by COST (European Cooperation in Science and Technology), and from the COVIRNA project which received funding from the European Union's Horizon 2020 Research and Innovation Programme under the grant agreement n°101016072. The Open Access fee of this article is covered by EU-CardioRNA COST Action CA17129, funded by COST.

**Funding**

L.B. is funded by the EU Horizon 2020 project COVIRNA (Grant Agreement # 101016072), the Spanish Ministry of Economy and Competitiveness of Science [PNS2016-76819-R]; the Carlos III Institute of Health [CIBERCV CB16/11/00411 and

Red Terapia Celular TerCel RD16/0011/0018] cofounded by FEDER; and the Fundación Investigación Cardiovascular-Fundación Jesus Serra.

E.L.R. is funded by the CardioVasculair Onderzoek Nederland (CVON) EARLY-HFPEF-2015 consortium (Dutch Heart Foundation).

A.J. is funded by a Horizon 2020 Marie Skłodowska-Curie Action (H2020-MSCA-IF-EF-ST 2019 Grant: 893435).

I.C. is funded by the EU Horizon 2020 project COVIRNA (Grant Agreement 101016072).

L.D.W acknowledges support from the Netherlands CardioVascular Research Initiative: the Dutch Heart Foundation, Dutch Federation of University Medical Centers, ZonMW and the Royal Netherlands Academy of Sciences (CVON2017-ARENA PRIME). L.D.W. was further supported by ERC Consolidator Grant 311549 CALMIRS, a VICI award 918-156-47 from NWO and a Marie Skłodowska-Curie grant agreement No 813716.

P.F. is funded by the National Research, Development and Innovation Office of Hungary (Research Excellence Program - TKP, National Heart Program NVKP 16-1-2016-0017, Investment into the Future – Covid-19 project at Semmelweis University); the Higher Education Institutional Excellence Program of the Ministry of Human Capacities in Hungary, within the framework of the Therapeutic Development thematic program of the Semmelweis University; EU Horizon 2020 projects COVIRNA (Grant #101016072) and CRYSTAL3 (MSCA-RISE Project #101007931).

M.G. is funded by the EU Horizon 2020 grants SCIENCE (643478), CRESPACE (732170) and ReGenHeart (731532).

M.H. is funded by Eurostars Grant no. 871562 and FFG Early Stage no. 874078.

M. L. is funded by the EU Horizon 2020 project COVIRNA (Grant Agreement 101016072) and the Slovenian Research Agency (research core funding No. P2-0209).

F.M. is funded by the Italian Ministry of Health, (“Ricerca Corrente and 5 x 1000), the Telethon Foundation (# GGP19035A) and AFM-Telethon grant (# 23054) and EU Horizon 2020 projects COVIRNA (Grant #101016072).

T.T. is funded by the EU Horizon 2020 project Cardioregenix.

Y.D. is funded by the EU Horizon 2020 project COVIRNA (Grant Agreement # 101016072), the National Research Fund (grants # C14/BM/8225223, C17/BM/11613033 and COVID-19/2020-1/14719577/miRCOVID.), the Ministry of Higher Education and Research, and the Heart Foundation – Daniel Wagner of Luxembourg.

**Author contributions**

L.B. and Y.D. designed the structure and content of the paper. All authors significantly contributed to the drafting of different sections of the paper. All authors reviewed, commented and accepted the final version of the manuscript.
